# A novel procedure for statistical inference and verification of gene regulatory subnetwork

**DOI:** 10.1186/1471-2105-16-S7-S7

**Published:** 2015-04-23

**Authors:** Haijun Gong, Jakob Klinger, Kevin Damazyn, Xiangrui Li, Shiyang Huang

**Affiliations:** 1 Department of Mathematics and Computer Science, Saint Louis University, 220 N Grand Blvd, 63103 St. Louis, MO, USA

**Keywords:** Gene regulatory network, statistical inference, dynamic Bayesian network, weighted model checking

## Abstract

**Background:**

The reconstruction of gene regulatory network from time course microarray data can help us comprehensively understand the biological system and discover the pathogenesis of cancer and other diseases. But how to correctly and efficiently decifer the gene regulatory network from high-throughput gene expression data is a big challenge due to the relatively small amount of observations and curse of dimensionality. Computational biologists have developed many statistical inference and machine learning algorithms to analyze the microarray data. In the previous studies, the correctness of an inferred regulatory network is manually checked through comparing with public database or an existing model.

**Results:**

In this work, we present a novel procedure to automatically infer and verify gene regulatory networks from time series expression data. The dynamic Bayesian network, a statistical inference algorithm, is at first implemented to infer an optimal network from time series microarray data of S. cerevisiae, then, a weighted symbolic model checker is applied to automatically verify or falsify the inferred network through checking some desired temporal logic formulas abstracted from experiments or public database.

**Conclusions:**

Our studies show that the marriage of statistical inference algorithm with model checking technique provides a more efficient way to automatically infer and verify the gene regulatory network from time series expression data than previous studies.

## Introduction

Advancement of DNA microarray technology and next generation sequencing technique have revolutionized the molecular biology, making it possible for biologists to measure and collect thousands of genes' expression levels simultaneously, efficiently and precisely in one experiment. Computational analysis of genome-wide transcriptomics data will help us understand the regulatory components and mechanisms underlying some diseases. These explosively growing amount of highdimensional gene expression data can be divided into two types: static and time series. The static expression data are assumed to be independently and identically distributed (IID), and many statistical inference algorithms [1-8] have been developed to identify key genetic signatures and signaling pathways that are frequently altered in some diseases. Gene regulatory network plays a critical role in the cell's proliferation and differentiation, so, a comprehensive understanding of gene regulatory network (GRN) and regulatory components will help discover some drug targeted genes in cancer and other diseases. Computational biologists have proposed a variety of methods, for example, the Boolean networks [9] and differential equations [10], to study the gene regulatory network. Friedman and other researchers [11,12] developed and applied discrete and continuous Bayesian networks (BN) with linear regression and non-parametric regression to infer gene regulatory networks. The BN approach could identify the causal relationships between different genes to some degree. However, it cannot construct cyclic networks and this method is unable to handle the temporal aspect of time-series data. But the feedback loops (cyclic pathways) are prevalent in the gene regulatory networks and signaling pathways.

The time series gene expression data can provide abundant information regarding the dynamic and temporal behaviors of biological system, which can not be handled by Bayesian network method. Dynamic Bayesian network (DBN) [13-16] is a promising alternative which has been proposed to construct GRN with feedback loops from time-series expression data. DBN has attracted a lot of attention from numerous bioinformatics researchers, and different DBN based approaches and tools were developed to increase accuracy and reduce computational time. An extended expectation-maximisation (EM) algorithm [17] was proposed to estimate the parameters in the DBN model. However, the DBN method has some limitations, for example, it is very sensitive to the choice of data discretization. Moreover, the deduction of the "activation" or "inhibition" relationship between different genes is not easy and accurate, so, the inferred optimal network might not be a correct one. Recently, Liang *et al.*'s work [18] proposed a network and community identification (NCI) method to infer multiple signed subnetworks from gene expression data by incorporating community structure information.

Without verification or validation, the inferred regulatory networks can not help us correctly understand the mechanism in the cell cycle. Another limitation in the previous studies is, the correctness or verification of the inferred networks is manually checked by comparing with public database (KEGG, GO, GenMAPP, etc) or existing/known models. This verification procedure is only good for small and already-known network "inference" and "verification". However, the signaling pathway or regulatory network is complex due to the excessive number of components and interactions, it is not realistic and efficient to use traditional methods to manually verify or analyze large networks. An intelligent verification technique called Model Checking [19] has been successfully applied for the verification of complex systems, including the hardware (e.g., CPU) and software (aerospace control software) systems. Recently, we applied this technique to study some complex biological networks [20-25]. Model Checking is the process of determining whether or not a given system *M *satisfies a desired temporal logic formula *ψ*, denoted by *M *╞ *ψ*. Our previous work proposed and applied different model checking techniques, including statistical model checking [21,22], synchronous symbolic model checking [23-25], asynchronous model checking technique [26] and probabilistic model checking [27], to formally verify some given stochastic, boolean, and discrete-value models of signaling pathways in the cancer cells. The model checkers automatically and exhaustively search the state space to verify some desired temporal logic formula, and it can check up to 10^100 ^possible states.

In this work, we proposed a novel inference and verification procedure, which marries the dynamic Bayesian network inference algorithm with a powerful model checking technique, to analyze time course microarray data. We will first briefly introduce the dynamic Bayesian network inference with Java objects (Banjo) [28] method developed in Hartemink's group [29] and apply it to infer optimal gene regulatory networks from time-series expression data. Then, we proposed a novel weighted symbolic model checking technique (weighted SMV) to automatically verify or falsify the inferred weighted networks or models through checking some temporal logic formulas abstracted from experiments. Finally, we apply Banjo and weighted SMV to analyze time-series microarray data and reconstruct gene regulatory subnetwork of yeast.

## Methods

### Dynamic Bayesian network inference

Probabilistic graphical model describes each node in the network by a random variable, and the directed edge represents a conditional dependence between two variables. Therefore, gene regulatory network can be graphically represented by a joint distribution of all random variables over time. The time series gene expression (microarray) data, which consists of *p *genes measured at *n *different time points, can be described by an *n *× *p *matrix **X**. If **X_i _**= (*X*_*i*1_,..., *X*_*ip*_)*^T ^*is defined as a random variable vector (at time *i*), then, **x_i _**= (*x*_*i*1_,...,*x*_*ip*_)^*T *^corresponds to the values of a vector of *p *genes' expression measured at time *i *= 1, 2,..., *n*; that is, *x_ij _*represents an observation value of the random variable *X_ij _*(the *j*th gene's expression measured at time *i*). We adopt some conventions used in Kim *et al.*'s work [13].

**Figure 1:**
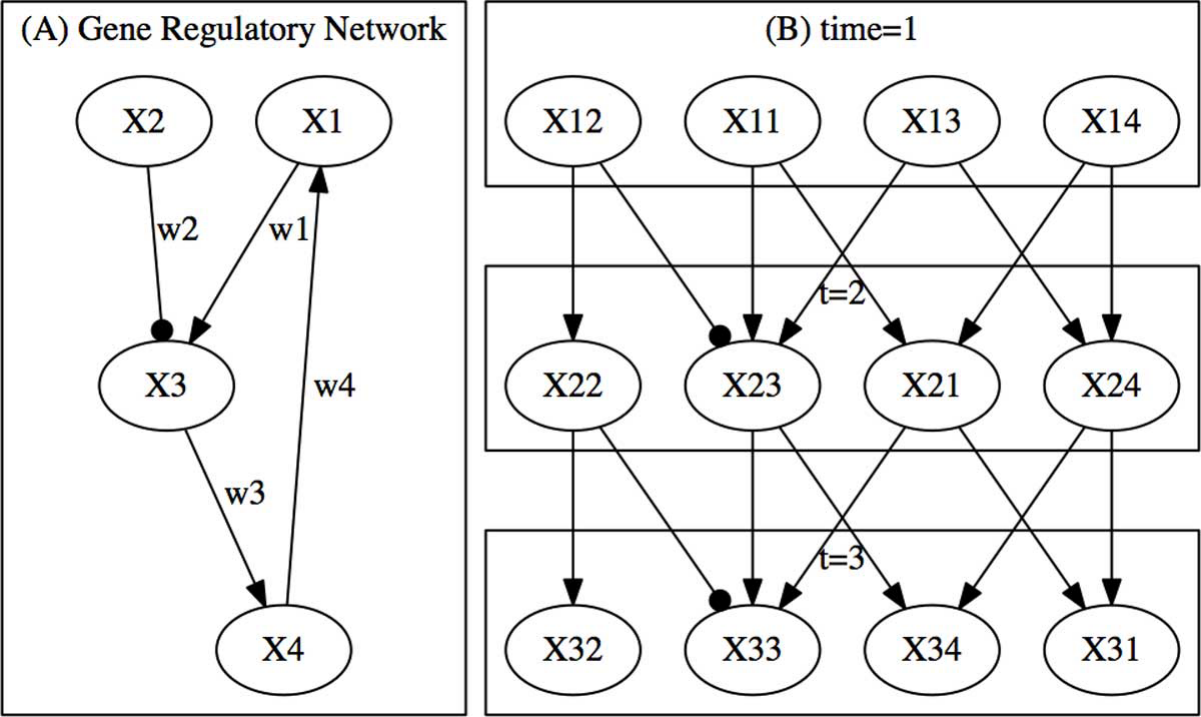
**Illustration of gene regulatory network (A) and dynamic Bayesian network (B)**. The gene regulatory network is composed of a feedback loop. Arrows represent activation, and circlehead arrows denote inhibition. The random variable *X_ij _*represents a gene *j *measured at time *i*.

Since the random variable vector **X_i _**is time dependent, the dynamic Bayesian network [13-15] assumes the genes' expression levels measured at time *i *are dependent on those at time *i *− 1 only which is illustrated in Figure 1. This assumption is also called first-order Markov chain. The joint probability distribution for the *n *× *p *random variables (or *n *vectors of random variables) can be written as

$$P\left(X_{1},X_{2},...,X_{n}\right)=P\left(X_{1}\right)P\left(X_{2}|X_{1}\right)...P\left(X_{n}|X_{n-1}\right).$$

We use *Par*(*X_ij_*) to denote the gene *j *(at time *i*)'s parents (at time *i − *1, an immediate previous time point), and also assume each gene (node) at time *I *is influenced by itself and its parent genes (nodes) at time *i − *1 only. Therefore, the conditional probability distribution can be expressed as

$$P\left(X_{i}|X_{i-1}\right)=P\left(X_{i1}|Par\left(X_{i1}\right)\right)...P\left(X_{ip}|Par\left(X_{ip}\right)\right).$$

**Figure 2:**
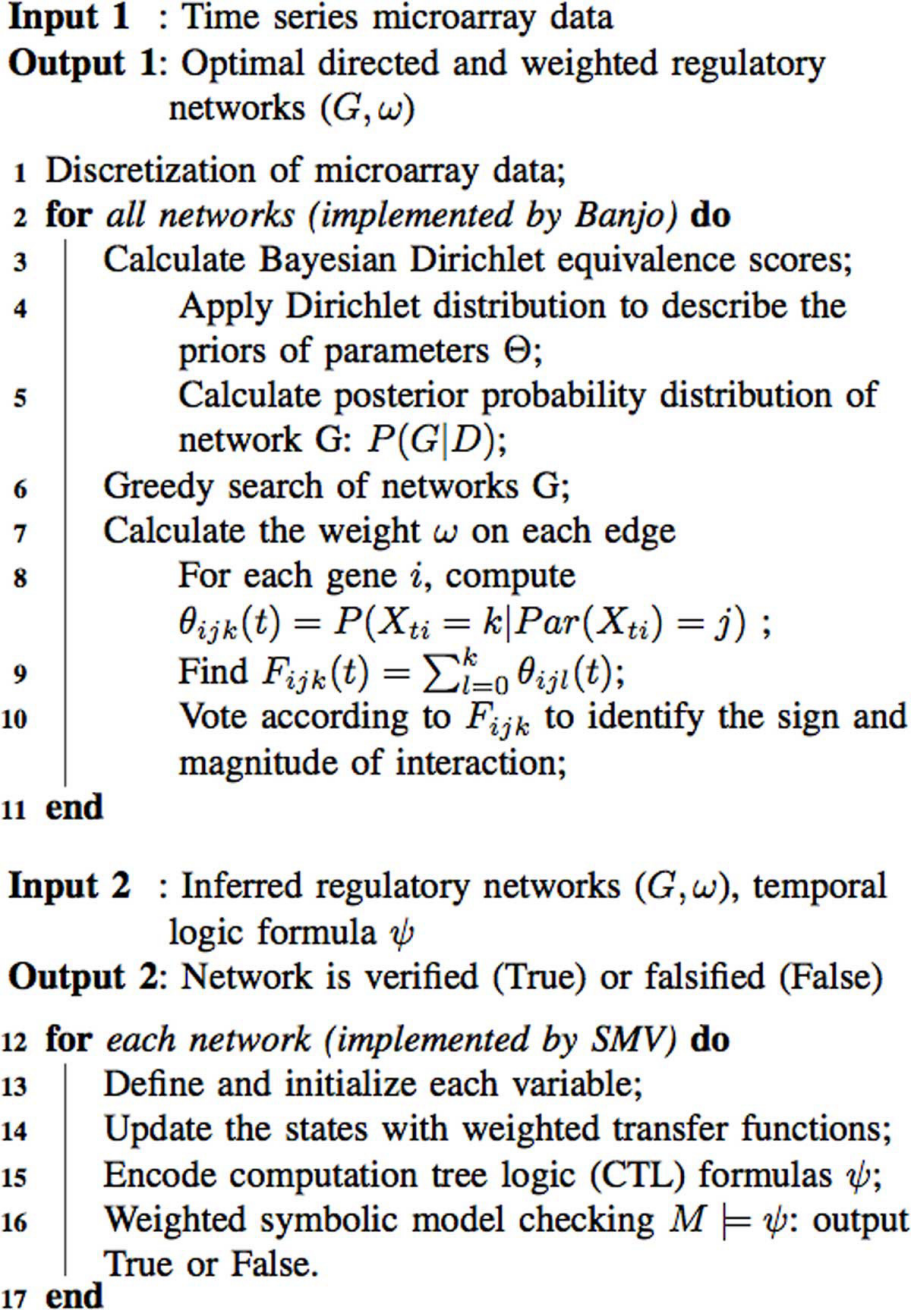
**Pseudocode of gene regulatory network inference and formal verification**. Part I describes the dynamic Bayesian network inference method implemeted by Banjo; part II describes the formal verification implemented by weighted symbolic model checker.

**Figure 3:**
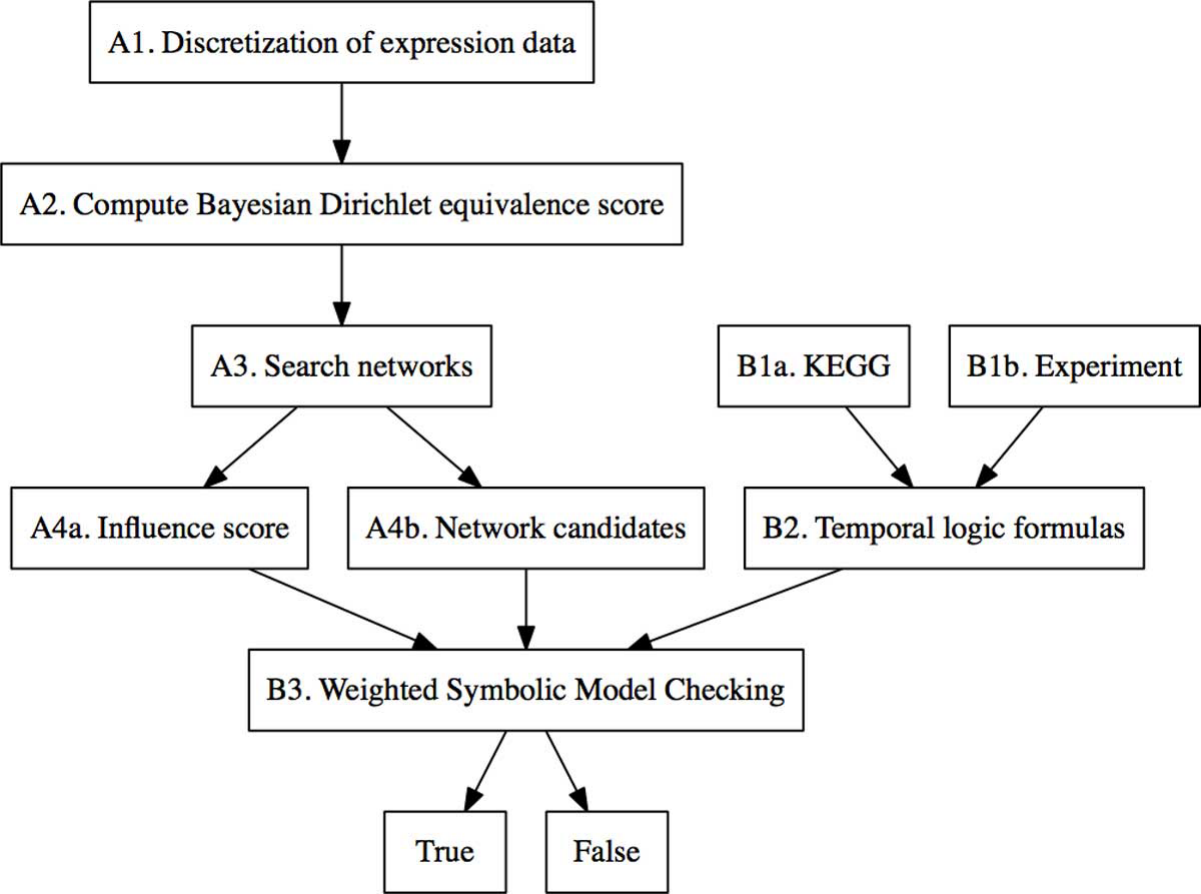
**Flowchart of gene regulatory network inference from time series microarray data and formal verification**. The dynamic Bayesian network inference (A1-A4) is implemented by Banjo, and the inferred network's verification (B1-B3) is implemented by the weighted symbolic model verifier (SMV).

Figure 2 and Figure 3 show the pseudocode and flowchart of the GRN inference and verification with dynamic Bayesian network learning method (implemented by Banjo) and weighted model checking technique (implemented by SMV model checker). First, the time series microarray data **D **are discretized into *k *levels {*l*_1_,..., *l_k_*}(*k *= 2, 3,...) using either quantile (*qk *) or interval discretization (*in*) methods [28]. Second, apply a Bayesian Dirichlet equivalence (BDe) scoring metric [30] to evaluate the goodness of each possible network. BDe scoring metric has been widely used as a criterion or score function in the regulatory network learning [13,15]. Then the idea is to find the posterior probability distribution of the possible networks **G**:

$$\begin{array}{ccc} P\left(G|D\right) & = & \frac{P\left(G,D\right)}{P\left(D\right)},\\ P\left(G,D\right) & = & \int P\left(G,D,\text{Θ}\right)d\text{Θ}\\ & = & \pi \left(G\right)\int f\left(D|G,\text{Θ}\right)\pi \left(\text{Θ}|G,\text{Λ}\right)d\text{Θ} \end{array}$$

The BDe score function is based on the assumption that the microarray data **D **is a multinomial sample, that is, **D**|**Θ **∼ *Multinomial*(**Θ**). BDe also assumes the parameters Θ are globally and locally independent and the priors of Θ, denoted by *π*(Θ|*G*, **Λ**), follow Dirichlet distribution with a hyperparameter vector **Λ**, that is, Θ|*G *∼ *Dirichlet*(**Λ**), which is a conjugate prior of multinomial distribution. The optimal network is selected according to the BDe scores which are dependent on *P *(*G*, **D**). Next, search all the possible optimal networks. Banjo allows two different search strategies, including the greedy search and simulated annealing algorithm proposed by Heckerman [31], which can output top *n *directed networks with highest scores, and it can also retain and average some highest scoring networks to produce a weighted consensus network [28].

Bayesian network inference with Java objects (Banjo) [28] can also compute the influence score (weight) [32] on each edge of the inferred optimal network. The value of influence score describes the relative magnitude of interactions, and its sign identifies the activation (a positive value) or inhibition (a negative value) relationship between two nodes (genes). The estimation of influence score [32] is dependent on the values of the conditional probability

$$\theta_{ijk}\text{(t)}=P\left(X_{ti}=k|Par\left(X_{ti}\right)=j\right),$$

which is the probability that gene *X_ti _*takes a value of *k *given its parent gene *Par*(*X_ti_*) takes a value of *j*; the cumulative distribution function $F_{ijk}\left(t\right)=\sum_{l=0}^{k}\theta_{ijl}\left(t\right)$, which describes the probability that gene *X_ti _*takes a value less than or equal to *k *given its parent gene takes a value of *j*; and a predefined voting system. If there is a high probability for the gene *X_ti _*to take a larger value given its parent's value increases, then, the voting system [32] in the Banjo will increase the positive vote by one; else, the negative vote will increase by one. If the influence score is close to 0, the sign of the edge can not be identified. Banjo can automatically implement the dynamic Bayesian network inference algorithm to search for high-scoring probabilistic graphical models, output the optimal networks and calculate the (signed) influence scores or weights. The interested reader could refer to [28,32] for details.

The dynamic Bayesian network implemented with Banjo can infer the high-scoring gene regulatory networks based on the BDe metrics, however, this algorithm is sensitive to the data discretization methods. Moreover, in many cases, the inferred optimal network might not be a correct one based on different scoring functions. Which model is closest to the truth in the biological system? Previous studies validate the inferred network through manually comparing with the public database or known models. The manual verification method is not realistic for the large or unknown network verification. The most innovative aspect of the proposed procedure in Figure 2 is the marriage of dynamic Bayesian network inference algorithm with formal verification technique, called weighted symbolic model checking (Part 2 of pseudocode in Figure 2), which can automatically verify the network through checking some temporal logic formulas abstracted from the experiments or public database. Next, we will introduce a powerful model checking technique and apply it to formally verify the inferred regulatory networks.

### Weighted symbolic model checking

A network or model can be described as a Kripke structure [19,20]* M *= (*S*, *s*_0_, *R*, *L*), representing a finite-state concurrent system with the initial state *s*_0 _∈ *S*, states transition relation *R *, and a labeling function *L*. Given a model or concurrent system, we expect it to satisfy some desired property. So, model checking, a formal verification technique [19], is the process of determining whether or not a given model *M *satisfies the desired property, which is expressed as a temporal logic formula *ψ*, denoted by *M *╞ *ψ*. During formal verification, model checkers can search the state space of concurrent system exhaustively to find all states that satisfy the formula *ψ*. If the property is satisfied, model checker will output "True"; else, it will output "False" with a counterexample sequence that falsifies *ψ*. Model checking of hardware and software systems has been very successful in the past three decades. Recently, we proposed different (probabilistic, statistical, symbolic, synchronous and asynchronous) model checkers to formally investigate the complex signal transduction networks in the cancer cells [20-24,26].

The desired properties describing some existing wet lab experimental results or phenomena are expressed in a high-level, expressive language - Computation Tree Logic (CTL) formula *ψ*. On the computation tree, the root represents an initial state, the branches and leaves represent all possible sequences of state transitions (paths) from the root [19]. CTL formula *ψ *is composed of path quantifiers which describes the branching structure in the computation tree: **A **(for all paths), **E **(there exists some path); temporal operators describing properties on a path through the tree: **X **(next time), **F **(in the future), **G **(globally), **U **(until), **R **(release); and Boolean logic connectives (| (or), & (and), *→ *(implies)). In the CTL formula, the temporal operator must be immediately preceded by a path quantifier [19]. Similar to our previous work [19,20,26], we will use (**AX, EX, AG, EG, AF, EF**) to construct CTL formulas for the verification of gene regulatory network. For example, **AG***φ *means *φ *is globally true on all paths; **EF***φ *means *φ *holds at some state in the future on some path. More interesting CTL operators and formulas have been discussed in Clarke *et al*.'s book [19].

Given a Kripke structure *M *, the state formula and path formula are represented by *ψ *and *φ *respectively in CTL syntax, and a path *π *is defined as an infinite sequence of states, *π *= *s*_0_, *s*_1_,..., where *s*_0 _is an initial state. We use *π_i _*to denote the suffix of *π *starting at state *s_i_*, and *M, π *╞ *φ *denotes the path *π *satisfies the path formula *φ*. The semantics of CTL have been defined in [19], below (Table 1) we list some semantics that are used in this work:

**Table 1:** 

*M*, *s *╞ ! *ψ*	iff *M*, *s *╞ *ψ *does not hold;
*M*, *s *╞ *ψ*_1 _&*ψ*_2_	iff *M*, *s *╞ *ψ*_1 _and *M, s *╞ *ψ*_2_;
*M*, *s *╞ *ψ*_1 _| *ψ*_2_	iff *M*, *s *╞ *ψ*_1 _or *M, s *╞ *ψ*_2_;
*M*, *π *╞ **X***ψ*	iff *M, π*^1 ^╞ *ψ*;
*M*, *π *╞ **F***ψ*	iff there exists a *k ≥ *0, such that *M, π^k ^*╞ *ψ*;
*M*, *π *╞ **G***ψ*	iff for all *k *≥ 0, *M*, *π^k ^╞ ψ*;
*M*, *s *╞ **A***φ*	iff for every path *π *from *s*, *M, π *╞ *φ*;
*M*, *s *╞ **E***φ*	iff there exists a path *π *from *s*, such that, *M*, *π *╞ *φ*,

The interested readers could refer to the book [19] and our recent work [20] for details regarding the syntax and semantics of CTL logic.

Symbolic Model Verifier (SMV) [33] is a popular formal verification tool encoded by ordered binary decision diagram [34], and the state transition relation is implicitly represented by a Boolean function. SMV can verify (output "True") or falsify (output "False" with a counterexample) a desired CTL formula *ψ *through automatically and exhaustively searching the state transition system *M*. Our recent studies [20,23-25,27] proposed both synchronous and asynchronous symbolic model checkers to study the Boolean and discrete value models of signaling pathways. These studies are based on the *unweighted *model checking, that is, the interaction between two nodes is represented by an unweighted edge.

**Figure 4:**
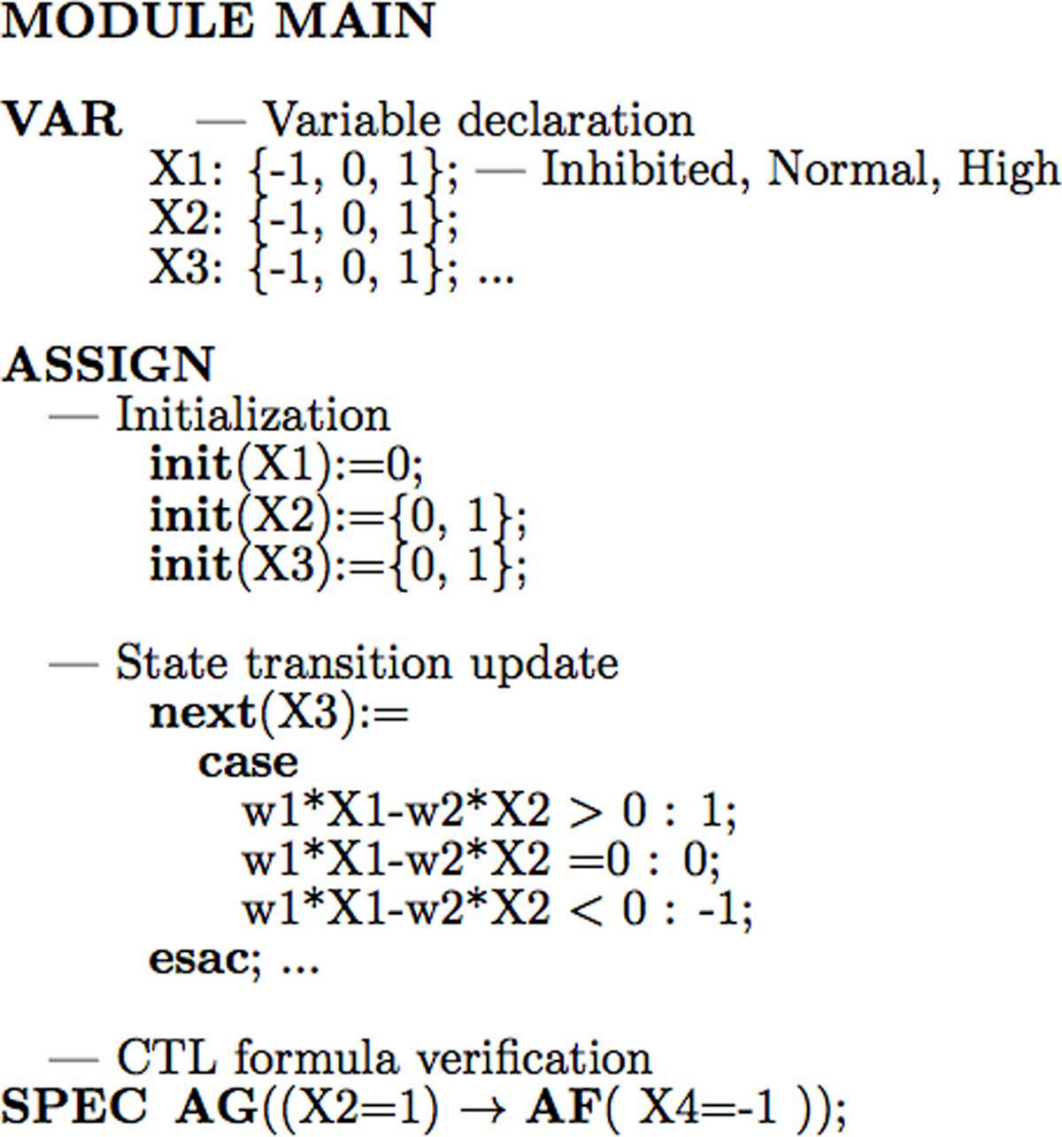
**Illustration of weighted symbolic model checking of the regulatory network in Fig**. 1. The state transition update is dependent on the modified influence score (weight *w_i_*) calculated by Banjo.

Next, we will propose a *weighted *symbolic model checking method (Part 2 of pseudocode in Figure 2) which is an extension of the unweighted model checker. Figure 4 illustrates some weighted SMV model checking code and CTL formulas for the verification of gene regulatory network given in Figure 1. The grammar of SMV code is similar to the unweighted SMV program [20,26], and both start with "MODULE MAIN". All the variables are declared with the keyword "VAR", and initialized with "init" under the keyword "ASSIGN". However, in the weighted SMV code, the state transition update of each variable (e.g., X3) is not only dependent on its parents' states (e.g., X1, X2), but also influenced by the strength of interactions, that is, the influence score or weight (e.g., *w*_1_, *w*_2_). The value of influence score calculated by Banjo [32] ranges from −1 to 1, which describes the sign and magnitude of interaction between two genes. Since the weighted SMV model checker does not allow floating point numbers, all the influence scores will be converted to integers (modified weights) first before formal verification. The CTL formula which abstracts the experimental phenomenon or public database is encoded with the keyword "SPEC". For example, the statement "SPEC AG(*X*2 = 1 *→ *AF(*X*4 = −1))" means, overexpressed *X*2 will eventually inhibit *X*4's expression on all paths. The weighted SMV model checker will automatically verify all the CTL formulas (encoded by SPEC), and find the best model which satisfies all or most of the properties based on existing experimental evidence.

## Results and discussion

In this section, we will apply the dynamic Bayesian network inference and weighted symbolic model checking methods proposed in Figure 2 to infer and verify gene regulatory subnetworks from time series microarray data of yeast.

The time series microarray data of *Saccharomyces cerevisiae *collected by Spellman *et al*. [35] has been studied by many researchers using different inference algorithms. The data were measured and collected from the yeast cultures synchronized by three independent methods: alpha factor arrest, elutriation, and arrest of a cdc15 temperature-sensitive mutant, which contain 16, 25 and 14 time points. A full description and complete data sets are available at [36]. The Banjo setting code, microarray data and weighted SMV code developed for this work are available at [37].

**Figure 5:**
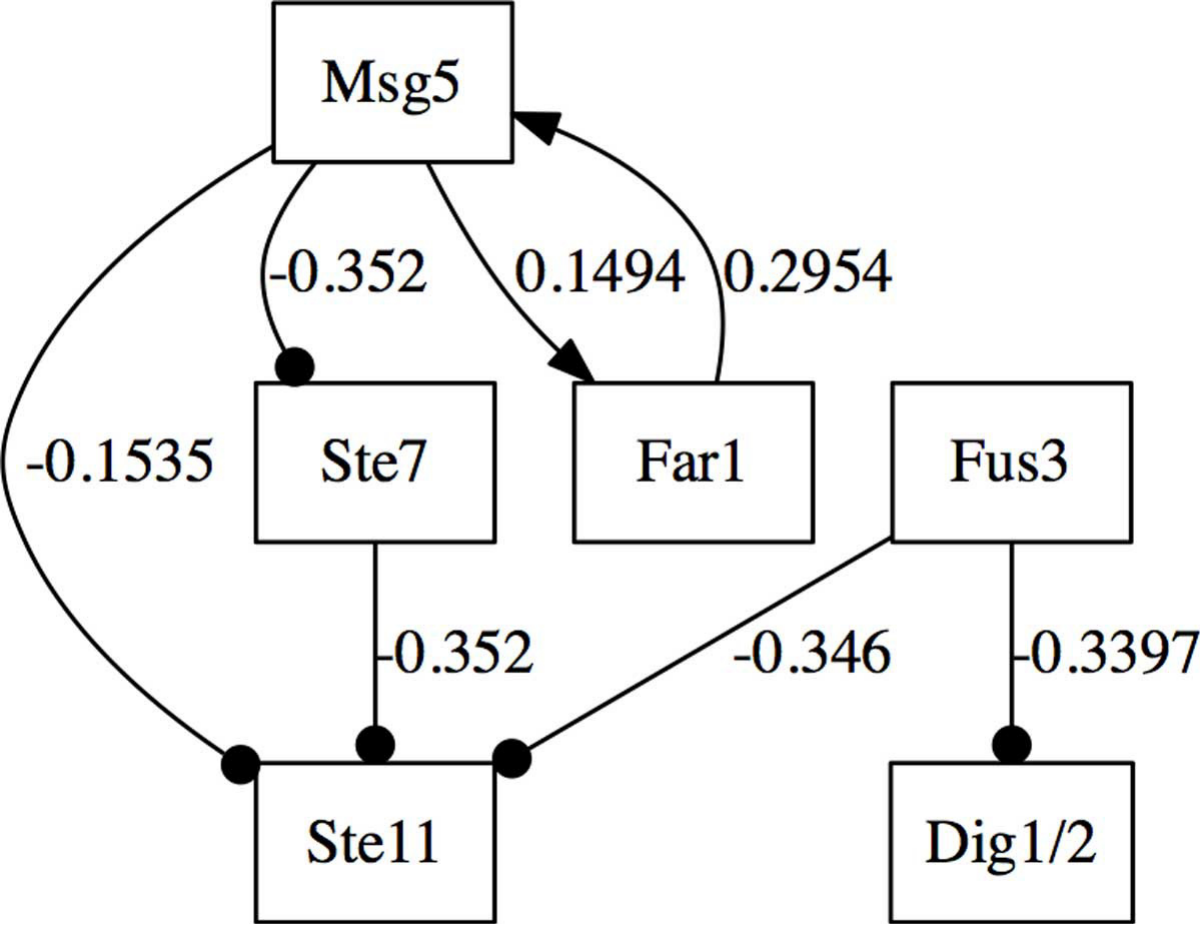
**An optimal subnetwork of MAPK pathway inferred by Banjo**. The optimal network is inferred based on *i*2 interval discretization method. The directed and circlehead arrows represent activation and inhibition respectively, the value on each edge is influence score or weight describing the interaction between two nodes.

We will first infer and verify a small network of MAPK signaling pathway which plays an important role in the cell cycle. We focused on the subnetwok around Fus3 which contains 8 genes (Ste20, Ste11, Ste7, Fus3, Dig1/2, Ste12, Far1, Msg5), while, Dig1/2 denotes the mean value of Dig1 and Dig2 in our analysis. Figure 5 shows an inferred optimal network which is composed of 6 genes based on *i*2 interval discretization (two discrete states) method in Banjo. The weighted symbolic model checker will be applied to formally verify or falsify this optimal network.

In Table 2 we summarize four CTL formulas abstracted from experiments and KEGG that MAPK pathway should satisfy. All the genes can take three possible states: inhibited (−1), normal (0), or activated (1), and they are initially set to be either 0 or −1 with a probability. Formula P1 is checking, if Fus3 is activated, Dig1/2 will be inhibited immediately in the next step (AX) on all paths. Our studies infer and verify that Fus3 is a direct inhibitor of Dig1/2. P2 means, it is globally true (AG) that Ste11 (MAPKKK)'s activation will immediately activate its downstream gene Ste7 (MAPKK) on all paths. P3 and P4 are checking whether or not Msg5 or Ste7's activation will finally inhibit or promote the transcription of Fus3 or Far1, cell cycle regulatory genes, respectively. The weighted SMV verified the formulas P1 and P3, but falsified P2 and P4. That is, the inferred network does not satisfy all the desired properties. So, this optimal network candidate inferred by Banjo is falsified by the weighted SMV model checker, which is also confirmed by the KEGG database. If some property is falsified, SMV model checker will also output a counterexample to demonstrate why this network is wrong, and help us refine the inferred network.

**Table 2:** List of CTL formulas related to MAPK pathway in Figure 5 and verification results

	CTL Formula	Result
P1	Fus3 = 1 → AX(Dig1/2 = -1)	True

P2	AG(Ste11 = 1 → AX(Ste7 = 1))	False

P3	Msg5 = 1 → AF(Fus3 = -1 & Far1 = -1 & Dig1/2 = 1)	True

P4	AG((Ste7 = 1 → AF(Fus3 = 1)) & (Fus3 = 1 → AF(Ste7 = 1)))	False

**Figure 6:**
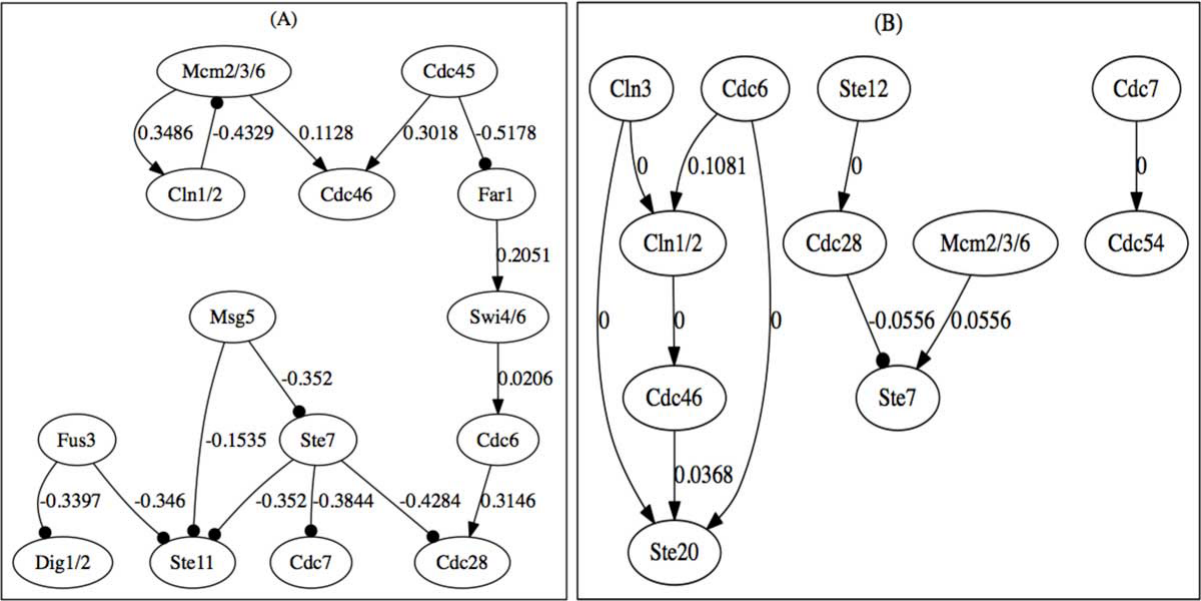
**Two optimal subnetworks of cell cycle inferred by Banjo**. (A) and (B) are inferred optimal networks based on the *i*2 interval discretization and *q*2 quantile discretization methods respectively.

Next, we will apply our methods to infer and verify a cell cycle subnetwork (including the genes: ste20, ste11, ste7, msg5, ste12, dig12, fus3, far1, cdc6, cdc7, cdc20, cdc28, cdc45, cdc46, cdc54, cln1/2, cln3, clb5/6, mcm2/3/6, swi4/6). Partial pathway has been registered in KEGG. Similar to MAPK pathway inference, we will use the mean values for some genes from the same family (e.g., mcm2, mcm3, mcm6) in the data analysis. Figure 6 shows two inferred candidates of "optimal" subnetworks based on interval (*i*2, Figure 6A) and quantile (*q*2, Figure 6B) discretization methods. The difference between these two "optimal" networks demonstrates that this inference algorithm is very sensitive to the choice of data discretization methods. Both figures in Figure 6 are weighted and directed, however, some weights on the Figure 6B are 0s, which means that, Banjo can not identify the signs (activation or inhibition) of these interactions. Next we will apply weighted SMV to verify the optimal network shown in Figure 6A.

Table 3 summarizes the verification results of some desired temporal logic formulas (Q1-Q4 are same as P1-P4 in Table 2) for the inferred *i*2 "optimal" network in Figure 6A. In the yeast, SWI4 regulates the transcription of Cln1 (property Q6), and Cdc28 is a downstream gene regulated by the MAPK pathway (Q7) [38]. SMV model checker verified Q6 but falsified Q7, which indicates a misconnection between Cdc28 and MAPK pathway during the network inference made by Banjo. Property Q9, which describes an oscillation behavior in the yeast, is also verified to be true by SMV model checker. So, 6 out of 9 properties are satisfied by the inferred network. Since the microarray data contains a small number of time points and a lot of measurement noise, we can not expect the inferred "optimal" networks to be completely correct. However, the model checking technique in this work can help identify the best optimal network which satisfies all or most temporal logic properties from all the possible candidates of inferred networks.

**Table 3:** CTL formulas related to cell cycle subnetwork in Figure 6A and verification results

	CTL Formula	Result
Q1:	Fus3 = 1 → AX(Dig1/2 = −1)	True

Q2:	AG(Ste11 = 1 → AX(Ste7 = 1))	False

Q3:	Msg5 = 1 → AF(Fus3 < 0 & Far1 < 0 & Dig1/2 = 1)	True

Q4:	AG((Ste7 = 1 → AF(Fus3 = 1)) & (Fus3 = 1 → AF(Ste7 = 1)))	False

Q5:	AG((Swi4/6 = 1 → AF(Cdc6 ≥ 0)))	True

Q6:	AG((Swi4/6 = 1 → AF(Cln12 ≥ 0)))	True

Q7:	AG((Cdc45 = 1 | Msg5 = 1 → EF(Cdc28 = 1 & Mcm2/3/6 ≥ 0)))	False

Q8:	EG((Swi4/6 = 1 → EF(Cdc28 ≤ 0)))	True

Q9:	AG((Mcm2/3/6 = 1 → AF(Cln12 = 1)) & (Cln12 = 1 → AF(Mcm2/3/6 ≤ 0)))	True

## Conclusions

A comprehensive understanding of the signaling pathways or gene regulatory networks will advance our knowledge in molecular biology. Network reconstruction from high-dimensional microarray data can help researchers to investigate the crosstalk of different pathways and develop effective multi-gene targeted treatments for some diseases, e.g., cancer and neurodegenerative diseases. Previous studies develop different statistical inference algorithms [13,14,39] to reconstruct gene regulatory network from time series expression data. The validation of inferred networks is implemented manually by comparing with public database or existing models, and, normally, a quantitative comparison is used to evaluate the superiority of a new approach [40,41]. In this work, we proposed a novel procedure, which integrates the dynamic Bayesian network inference algorithm with formal verification technique implemented by Banjo and weighted SMV model checker respectively, to analyze time series gene expression data. The dynamic Bayesian network inference algorithm implemented by Banjo could infer optimal networks of highest scores with directed and weighted edges, however, this method is sensitive to the choice of data discretization methods. The weighted symbolic model checker will exhaustively search the state space to verify or falsify these network candidates through checking some desired temporal logic formulas. Compared with previous studies, the proposed procedure can automatically infer, verify or falsify a biological network based on existing experiments, so it has advantages in the large network inference and verification. The goodness of the verified network will be dependent on not only the learning scores, but also the number of verified temporal logic formulas. One of the key issues in the model checking procedure is the quantity and also quality of the desired temporal logic formulas, which can be abstracted directly from existing experimental results or public database. The more temporal properties we have, the more constrains we can impose on the inferred network candidates. Currently, the inferred regulatory networks are manually encoded into SMV program for model checking. Our future work will build a bioinformatics infrastructure which integrates statistical inference algorithms with different model checkers in a unified framework to automatically infer, encode network candidates into SMV program, and formally verify the inferred gene regulatory networks to select the best models.

## Competing interests

The authors declare that they have no competing interests.

## Authors' contributions

HG proposed the study, HG, JK, KD prepared the computational code, HG, JK, KD, XL, SH analyzed the results, HG wrote the manuscript. All authors approved the final manuscript.

## Declarations

Publication of this article was funded by the Saint Louis University to HG.

This article has been published as part of *BMC Bioinformatics *Volume 16 Supplement 7, 2015: Selected articles from The 11th Annual Biotechnology and Bioinformatics Symposium (BIOT-2014): Bioinformatics. The full contents of the supplement are available online at http://www.biomedcentral.com/bmcbioinformatics/supplements/16/S7.

## References

[B1] Statnikov A Aliferis CF Tsamardinos I Hardin D Levy S A comprehensive evaluation of multicategory classification methods for microarray gene expression cancer diagnosis Bioinformatics 2005 21 631 643 10.1093/bioinformatics/bti033 15374862

[B2] Luan Y Li H Group additive regression models for genomic data analysis Biostatistics 2008 9 100 113 10.1093/biostatistics/kxm015 17513311

[B3] Wu TT Chen YF Hastie T Sobel E Lange K Genomewide association analysis by lasso penalized logistic regression Bioinformatics 2009 25 714 721 10.1093/bioinformatics/btp041 19176549 PMC2732298

[B4] Ma S Song X Huang J Supervised group lasso with applications to microarray data analysis BMC Bioinformatics 2007 8 60 76 10.1186/1471-2105-8-60 17316436 PMC1821041

[B5] Wu TT Wang S Doubly regularized cox regression for high-dimensional survival data with group structures Statisstics and Its Interface 2013 6 175 186 10.4310/SII.2013.v6.n2.a2

[B6] Yuan M Lin Y Model selection and estimation in regression with grouped variables J R Statist Soc B 2006 68 49 67 10.1111/j.1467-9868.2005.00532.x

[B7] Wu TT Gong H Clarke EM A transcriptome analysis by lasso penalized cox regression for pancreatic cancer survival Journal of Bioinformatics and Computational Biology 2011 9 63 10.1142/S0219720011005744 22144254

[B8] Gong H Wu TT Clarke EM Pathway-gene identification for pancreatic cancer survival via doubly regularized cox regression BMC Systems Biology 2014 8 10.1186/1752-0509-8-S1-S3 PMC4080266 24565114

[B9] Akutsu T Miyano S Kuhara S Inferring qualitative relations in genetic networks and metabolic pathways Bioinformatics 2000 16 727 734 10.1093/bioinformatics/16.8.727 11099258

[B10] Chen T He H Church G Moeling gene expression with differential equations Pacific Symposium on Biocomputing 1999 29 40 10380183

[B11] Friedman N Linial M Nachman I Pe'er D Using bn to analyze expression data J Comp Biol 2000 7 601 620 10.1089/106652700750050961 11108481

[B12] Imoto S Goto T Miyano S Estimation of genetic networks and functional structures between genes by using bn and nonparametric regression Pacific symposium on Biocomputing 2002 11928473

[B13] Kim S Imoto S Miyano S Inferring gene networks from time series microarray data using dynamic bayesian networks Briefings in Bioinformatics 2003 4 228 235 10.1093/bib/4.3.228 14582517

[B14] Friedman N Murphy K Russell S Learning the structure of dynamic probabilistic networks Prpceedings of the 14th conference on the uncertainty in artificial intelligence 1998

[B15] Ong I Glasner J Page D Modelling regulatoruypathways in e. coli from time series expression profiles Bioinformatics 2002 18 241 248 10.1093/bioinformatics/18.suppl_1.s241 12169553

[B16] Kim S Imoto S Miyano S Dynamic bayesian network and nonparametric regression for nonlinear modeling of gene networks from time series gene expression data BioSystems 2004 75 57 65 10.1016/j.biosystems.2004.03.004 15245804

[B17] Perrin B Ralaivola L Mazurie A Gene networks inference using dynamic bayesian networks Bioinformatics 2003 74 138 148 10.1093/bioinformatics/btg1071 14534183

[B18] Liang X Xia Z Zhang L Wu F Inference of gene regulatory subnetworks from time course gene expression data BMC Bioinformatics 2012 13 3 10.1186/1471-2105-13-3 22901088 PMC3372453

[B19] Clarke EM Grumberg O Peled DA Model Checking 1999 MIT Press

[B20] Gong H Analysis of intercellular signal transduction in the tumor microenvironment BMC Systems Biology 2013 7 Suppl 3 S5 10.1186/1752-0509-7-S3-S5 24555417 PMC3852214

[B21] Gong H Zuliani P Komuravelli A Faeder JR Clarke EM Analysis and verification of the HMGB1 signaling pathway BMC Bioinformatics 2010 11 Suppl 7 S10 10.1186/1471-2105-11-S7-S10 21106117 PMC2957678

[B22] Gong H Zuliani P Komuravelli A Faeder J Clarke E Computational modeling and verification of signaling pathways in cancer Proceedings of Algebraic and Numeric Biology, LNCS 2012 6479

[B23] Gong H Wang Q Zuliani P Lotze MT Faeder JR Clarke EM Symbolic model checking of the signaling pathway in pancreatic cancer Proceedings of the International Conference on Bioinformatics and Computational Biology (BICoB) 2011

[B24] Gong H Zuliani P Clarke E Model checking of a diabetes-cancer model3rd International Symposium on Computational Models for Life Sciences 2011

[B25] Gong H Wang Q Zuliani P Clarke E Formal analysis for logical models of pancreatic cancer50th IEEE Conference on Decision and Control and European Control Conference 2011

[B26] Gong H Feng L Computational analysis of the roles of er-golgi network in the cell cycle BMC Systems Biology 2014 8 Suppl 4 S3 10.1186/1752-0509-8-S4-S3 25522186 PMC4290691

[B27] Gong H Feng L Probabilistic verification of er stress-induced signaling pathways Proceedings of IEEE International Conference on Bioinformatics and Biomedicine 2014

[B28] Sladeczek J Hartemink A Robinson J Banjo: Bayesian network inference with java objects User Guide 2005

[B29] Banjo Software http://www.cs.duke.edu/~amink/software/banjo/

[B30] Heckerman D Geiger D Chickering D Learning Bayesian networks: The combination of knowledge and statistical data Machine Learning 1995 20 3

[B31] Heckerman D A tutorial on learning with bayesian networks Technical Report MSR-TR-95-06, Microsoft Research 1996

[B32] Yu J Smith V Wang P Hartemink A Jarvis E Advances to bayesian network inference for generating causal networks from observational biological data Bioinformatics 2004 20 3594 3603 10.1093/bioinformatics/bth448 15284094

[B33] McMillan KL PhD Thesis: Symbolic Model Checking an Approach to the State Explosion Problem 1992 Carnegie Mellon University

[B34] Bryant RE Graph-based algorithms for boolean function manipulation IEEE Tran. on Computers 1986 35 8 677 691

[B35] Spellman P Sherlock G Zhang M Comprehensive identification of cell cycle-regulated genes of the yeast saccharomyces cerevisiae by microarray hydridization Mol Biol Cell 1998 9 3273 3297 10.1091/mbc.9.12.3273 9843569 PMC25624

[B36] S. Cerevisiae Expression Data by Spellman http://downloads.yeastgenome.org/expression/microarray/

[B37] Computer Code http://cs.slu.edu/~gong/Banjocode.zip

[B38] Chai L Mohamad M A dynamic bayesian network-based model for inferring gene regulatory networks from gene expression data International Journal of Bio-Science and Bio-Technology 2014 6 41 52

[B39] Novikov E Barillot E Regulatory network reconstruction using an integral additive model with flexible kernel functions BMC Systems Biology 2008 2 8 10.1186/1752-0509-2-8 18218091 PMC2248159

[B40] R RS Ventura D Prince J Controlling for confounding variables in ms-omics protocol: why modularity matters Brief Bioinform 2014 15 5 768 70 10.1093/bib/bbt049 23894105

[B41] Smith1 R Ventura D Prince JT Novel algorithms and the benefits of comparative validation Bioinformatics 2013 29 1583 1585 10.1093/bioinformatics/btt176 23589651

